# Supplementary material for: CTIVA: Censored time interval variable analysis
Source: PLoS One. 2023 Nov 16;18(11):e0294513. doi: 10.1371/journal.pone.0294513 (PMC10653491; doi:10.1371/journal.pone.0294513)
Supplement: S1 Table — (DOCX) [file pone.0294513.s001.docx]

S1 Table. Comparison results of sensitivity and specificity at p-value 0.05.

| Data Type | Metric  Method | Sensitivity | Specificity |
| --- | --- | --- | --- |
| Categorical | CTIVA | 0.98$\pm$0.06 | 0.84$\pm$0.02 |
|  | Cox Event 1 | 0.89$\pm$0.07 | 0.27$\pm$0.05 |
|  | Cox Event 2 | 0.94$\pm$0.04 | 0.75$\pm$0.01 |
|  | Ignored | 0.61$\pm$0.15 | 0.92$\pm$0.02 |
|  | No Censor | 0.85$\pm$0.07 | 0.87$\pm$0.01 |
| Combined | CTIVA | 0.98$\pm$0.06 | 0.85$\pm$0.07 |
|  | Cox Event 1 | 0.89$\pm$0.07 | 0.25$\pm$0.01 |
|  | Cox Event 2 | 0.94$\pm$0.04 | 0.77$\pm$0.01 |
|  | Ignored | 0.61$\pm$0.15 | 0.92$\pm$0.01 |
|  | No Censor | 0.85$\pm$0.07 | 0.88$\pm$0.01 |

The dataset was sampled from an additive exponential distribution which is same as used in Table 1 and ANOVA test was implemented as statistical test.
