# Supplementary material for: CTIVA: Censored time interval variable analysis
Source: PLoS One. 2023 Nov 16;18(11):e0294513. doi: 10.1371/journal.pone.0294513 (PMC10653491; doi:10.1371/journal.pone.0294513)
Supplement: S2 Table — (DOCX) [file pone.0294513.s002.docx]

S2 Table. Comparison results of sensitivity and specificity at p-value 0.01 and p-value 0.1.

| p-value | Metric  Method | Sensitivity | Specificity |
| --- | --- | --- | --- |
| Categorical  p-value 0.01 | CTIVA | 0.98$\pm$0.06 | 0.89$\pm$0.03 |
|  | Cox Event 1 | 0.89$\pm$0.07 | 0.24$\pm$0.05 |
|  | Cox Event 2 | 0.94$\pm$0.04 | 0.79$\pm$0.01 |
|  | Ignored | 0.61$\pm$0.15 | 0.98$\pm$0.01 |
|  | No Censor | 0.85$\pm$0.07 | 0.93$\pm$0.02 |
| Categorical  p-value 0.1 | CTIVA | 0.98$\pm$0.06 | 0.90$\pm$0.09 |
|  | Cox Event 1 | 0.89$\pm$0.07 | 0.21$\pm$0.01 |
|  | Cox Event 2 | 0.94$\pm$0.04 | 0.83$\pm$0.01 |
|  | Ignored | 0.61$\pm$0.15 | 0.98$\pm$0.01 |
|  | No Censor | 0.85$\pm$0.07 | 0.94$\pm$0.01 |
| Combined  p-value 0.01 | CTIVA | 0.98$\pm$0.06 | 0.79$\pm$0.03 |
|  | Cox Event 1 | 0.89$\pm$0.07 | 0.31$\pm$0.05 |
|  | Cox Event 2 | 0.94$\pm$0.04 | 0.70$\pm$0.01 |
|  | Ignored | 0.61$\pm$0.15 | 0.87$\pm$0.01 |
|  | No Censor | 0.85$\pm$0.07 | 0.82$\pm$0.02 |
| Combined  p-value 0.1 | CTIVA | 0.98$\pm$0.06 | 0.80$\pm$0.06 |
|  | Cox Event 1 | 0.89$\pm$0.07 | 0.30$\pm$0.01 |
|  | Cox Event 2 | 0.94$\pm$0.04 | 0.72$\pm$0.01 |
|  | Ignored | 0.61$\pm$0.15 | 0.86$\pm$0.02 |
|  | No Censor | 0.85$\pm$0.07 | 0.83$\pm$0.01 |

The dataset was sampled from an additive exponential distribution which is same as used in Table 1 and ANOVA test was implemented as statistical test.
