# Supplementary material for: CTIVA: Censored time interval variable analysis
Source: PLoS One. 2023 Nov 16;18(11):e0294513. doi: 10.1371/journal.pone.0294513 (PMC10653491; doi:10.1371/journal.pone.0294513)
Supplement: Attachment — Submitted filename: Response to Reviewers.docx [file pone.0294513.s003.docx]

September 11, 2023

Dear Editor,

We greatly appreciate the valuable comments and helpful advices from the editors and reviewers. Enclosed please find the revised manuscripts with tracked changes titled as “Revised Manuscript with Track Changes”. We considered each comment carefully and revised the manuscript accordingly.

Below please check our point-by-point responses to the reviewers’ comments. The reviewers’ original comments are in blue and our responses are in black. The revisions in the manuscript are highlighted in yellow. We once again thank the valuable helps the editor and reviewers have given.

**Reviewer #1:**

Comments 1. Overall, the paper proposed a novel way to handle multiple censored datasets. The paper would be stronger if CTIVA can be compared with other state of the art methods such as survival analysis techniques or other machine learning methods.

Author’s response: We would like to express our appreciation for the valuable feedback provided by the reviewer. We have carefully considered their comments and have incorporated their suggestions into our manuscript.

Regarding the reviewer’s suggestion to compare the proposed CTIVA model with other state of art methods, we agree that this would help demonstrating the effectiveness of the proposed method. Therefore, we have added a comparison result of CTIVA model and Cox survival analysis model in the simulation result section.

As highlighted in the revised manuscript, the additional test results acquired through the Cox survival analysis is provided in the simulation result section. We have modified the Table 2 and 3 and Figure 1 and 2 to add the Cox survival analysis results [1]. We believe supplementing the data with the existing state of the art models would help emphasizing the effectiveness of the proposed method.

Comments 2. While the paper applies CTIVA to a real-world medical history dataset, the analysis is limited to a small subset of variables. It would be beneficial to include a more comprehensive analysis on larger real-world datasets to demonstrate the generalizability and robustness of CTIVA in different domains and data types.

Author’s response: Thank you for your valuable comment on our paper. We appreciate your suggestion to extend our method on larger real-world datasets. We have applied CTIVA in proteasome inhibitor bortezomib dataset provided by National Center for Biotechnology Information (NCBI) [2]. The application result of the proposed CTIVA in proteasome inhibitor bortezomib dataset is supplemented in Real Dataset Results section. The proposed CTIVA also demonstrated novel performance in new dataset finding the medical categorical variable; number of prior lines to be statistically significantly correlated with the time interval between pharmacogenomics progression date and death. The result of the CTIVA corresponds with the traditional medical studies about clinical trial outcome of proteasome inhibitor bortezomib patients [3].

**Reviewer #2:**

Comments 1. The concept of the work was clearly demonstrated but the data to support the concept was notably lacking. For example, the AUC was calculated and compared among multiple algorithms but explanation on how the data was derived was not clear and the statistic significance was missing. For example in Figure 1 and 2, what is the cutoff when deriving the AUC; what is statistical significance of each AUC; what is the sensitivity and specificity of each AUC; what is the significance when comparing three algorithms? It is suggested for authors to include more data in the supplementary information.

Author’s response: We appreciate the reviewer’s helpful comments in improving the manuscript. We have carefully considered their comments and have implemented their suggestion into our manuscript.

Regarding the reviewer’s suggestion to enhance the description about the AUC in simulation result section, we additionally provided the cutoff while driving the AUC; sensitivity and specificity of each AUC in the simulation result section. In specific, the cutoff value of AUC was set as 0.05 and the CTIVA showed superior results compared to baseline method in both sensitivity and specificity with the provided cutoff value. With the cutoff p-value 0.05 in categorical dataset, the average sensitivity of CTIVA is 0.96 while the average specificity is 0.84. Additionally, in combined dataset, the average sensitivity of CTIVA is 0.88 while the mean specificity is 0.83. These modifications are added in page 11, Simulation Results in Uncorrelated Dataset section and we have included detailed comparison in supplementary.

Comments 2. Certainly, as stated in the Data Availability section, there was a link. However, it requests a fee to access the data. This lack of access hindered my ability to fully comprehend and evaluate the authenticity of this study. The lack of data availability raises concerns about the reproducibility and reliability of the study's results.

Author’s response: Thank you for your acute point that data is not available. We have found out that demo data we used for the real-world test is only freely available for domestic users who are citizens of South Korea. The Cohort Demo dataset we have used for the experiment is open access for the domestic users but not freely available for foreign users and require fee for the access. We deeply apologize the inconvenience we have made due to the lack of double checking the data sharing policy of National Health Insurance Sharing Service of Republic of Korea for foreign users.

Due to the lack of data availability of the Cohort Demo dataset, we have committed proteasome inhibitor bortezomib dataset provided by NCBI in github. The source code that generates the simulation samples and applies the CTIVA with the provided dataset is also committed in the following link [<https://github.com/Insoo-K/CTIVA>].

Comments 3. The background provided in this work could benefit from a more comprehensive and specific review of related references. Distinguishing CTIVA from other existing methodologies, such as the competing risk regression model, would enhance the clarity and uniqueness of the approach.

Author’s response: We gradually appreciate the reviewer’s helpful suggestion in improving the manuscript by supplementing the related references in the manuscript. We have implemented the reviewer’s suggestion in the introduction section and inserted the paragraph in the manuscript as below.

Although, there exist survival analysis models that handle multiple events such as competing risk regression model, it only handles the correlated events. Therefore, method that handles multiple independent censored events, should be further investigated.

The proposed CTIVA handles multiple independent censored time to event data where competing risk regression model cannot be implemented.

**Reviewer #3:**

Comments 1. The background provided in this work could benefit from a more comprehensive and specific review of related references. Distinguishing CTIVA from other existing methodologies, such as the competing risk regression model, would enhance the clarity and uniqueness of the approach.

Author’s response: We gradually appreciate the reviewer’s valuable suggestion in improving the manuscript by demonstrating the uniqueness of CTIVA compared to existing methods. Our proposed CTIVA handles multiple independent censored time to event data where most of existing methods cannot handle. We have applied the reviewer’s suggestion in the introduction section and modified the manuscript as below.

Although, there exist survival analysis models that handle multiple events such as competing risk regression model, it only handles the correlated events. Therefore, method that handles multiple independent censored events, should be further investigated.

Comments 2. To improve the manuscript, the authors should include more detailed information in key sections. For instance, providing a thorough sensitivity analysis of different parameter settings in the simulation study would strengthen the validity of the findings. Additionally, clear descriptions of baseline models compared to CTIVA and the rationale behind their selection for comparison should be included.

Author’s response: We thank the reviewer’s helpful suggestion in enriching the manuscript. We have implemented the reviewer’s valuable feedback in the manuscript and inserted the additional sensitivity and specificity analysis in Simulation Results in Uncorrelated Dataset section and Supplementary. Also, we have added descriptions of baseline models in the Simulation Settings section as below.

The proposed CTIVA method was compared with various baseline methods for the performance verification. The baseline methods include naïve methods that do not consider the presence of censoring by ignoring the censoring or only considering the sample without censoring. In specific, the naïve method that ignores censoring considers the censored time event as actual observed event. Furthermore, we have also conducted the experiment with Cox Regression Model, the method that is dominantly used for handling the censored time to event data [4].

1.	Cox DR. Regression models and life‐tables. Journal of the Royal Statistical Society: Series B (Methodological). 1972;34(2):187-202.

2.	Mulligan G, Mitsiades C, Bryant B, Zhan F, Chng WJ, Roels S, et al. Gene expression profiling and correlation with outcome in clinical trials of the proteasome inhibitor bortezomib. Blood. 2007;109(8):3177-88.

3.	Boccadoro M, Morgan G, Cavenagh J. Preclinical evaluation of the proteasome inhibitor bortezomib in cancer therapy. Cancer cell international. 2005;5:1-9.

4.	Therneau TM, Grambsch PM. The cox model. Modeling survival data: extending the Cox model: Springer; 2000. p. 39-77.
