# Supplementary material for: CTIVA: Censored time interval variable analysis
Source: PLoS One. 2023 Nov 16;18(11):e0294513. doi: 10.1371/journal.pone.0294513 (PMC10653491; doi:10.1371/journal.pone.0294513)
Supplement: Attachment — Submitted filename: Response to Reviewers.docx [file pone.0294513.s004.docx]

October 16, 2023

Dear Editor,

We greatly appreciate the valuable comments and helpful advices from the editors and reviewers. Enclosed please find the revised manuscripts with tracked changes titled as “Revised Manuscript with Track Changes”. We considered each comment carefully and revised the manuscript accordingly.

Below please check our point-by-point responses to the reviewers’ comments. The reviewers’ original comments are in blue and our responses are in black. The revisions in the manuscript are highlighted in yellow. We once again thank the valuable helps the editor and reviewers have given.

**Reviewer #3:**

Comments 1. Tables 2 and 3 lack clear descriptions. In the context of sensitivity analysis, it would be helpful to know if there were any disparities in the results when various parameter hypotheses were considered. Additionally, some details about the simulation process, such as the distribution characteristics of the variables, would be valuable for a more comprehensive understanding. We anticipate that CTIVA will outperform the baseline methods across various parameter hypotheses and settings.

Author’s response: We greatly appreciate your constructive suggestions, which have significantly contributed to enhancing our manuscript. We have conducted an additional experiment verifying the effectiveness of CTIVA in various setting. Varying the cutoff p-value by 0.1 and 0.01, the proposed CTIVA still outperformed other benchmark methods in terms of sensitivity and specificity. Additionally, we have revised some value errors overlooked in Table S1. The detailed comparison results are demonstrated in Table S1 and S2 in Supplementary section. Furthermore, we have refined the manuscript by moving the Table 1, which analyzes data generation procedure, to Simulation Setting section while including the detailed explanation about datasets generated through simulation right behind the Table 1.

Comments 2. I would suggest the authors incorporate a more in-depth discussion regarding CTIVA, including the inherent limitations previously mentioned in the discussion section.

Author’s response: Thank you for your valuable comment on our paper. We appreciate your suggestion to supplement the inherent limitations of the proposed CTIVA in the discussion section of the manuscript. The limitations of the proposed method due the joint probability function estimation are described in the manuscript as written below.

Although, the CTIVA showed novel results in detecting correlated variables, some limitations exist in the proposed method. Since the proposed methods estimates the joint probability distribution with the Bayesian estimation, disparity between the actual distribution and estimated distribution that influences the prediction results might exist. Also, the proposed CTIVA relies the probability estimation on the convergence of the density estimation function. Therefore, due to the variety of the computing time of the function convergence, the computational cost of the proposed method might surge depending on the task.

**Reviewer #4:**

Comments 1. The work titled "Network estimation for censored time-to-event data for multiple events based on multivariate survival analysis" appears to be closely aligned with the themes of this manuscript. It would enhance the manuscript's depth if this work is introduced early on, preferably in the introduction. A comparative analysis with other existing methodologies, highlighting the advancements and distinctions of CTIVA in analyzing censored time-to-event data, would provide readers with a clearer understanding of the manuscript's contributions.

Author’s response: We greatly appreciate the reviewer’s valuable suggestion supplementing the manuscript by referring the previous study about multivariate survival analysis. We have introduced the previous study covering multivariate censored data in introduction section. The inserted paragraph is highlighted in manuscript with tracked changes as yellow. Furthermore, we have demonstrated the distinct advantage of the proposed CTIVA in handling categorical and combined variables. Specifically, we have inserted the following paragraph in the last paragraph of introduction section to notate the contribution of the CTIVA.

Moreover, CTIVA demonstrates practical advantages compared to previous methods. Our proposed method addresses challenges posed by diverse variable types, a facet not adequately tackled by prior studies. Specifically, the proposed method could be implemented in clinical trial and symptoms which could be considered as categorical variables. Thus, the proposed CTIVA has extended the solution of multivariate censored time-to-event dataset to more general fields.

Comments 2. While the manuscript provides a comprehensive list of references, there appears to be a reliance on older publications. Specifically, only 4 out of the 21 cited papers have been published in the last four years, with the most recent one dating back to 2021. To ensure the manuscript is grounded in the latest research, it would be beneficial to incorporate more contemporary references relevant to the topic.

Author’s response: We thank the reviewer’s helpful suggestion to enhance the manuscript by citing more contemporary references. We have reviewed recent literatures that highlight cutting-edge method for handling censored data in survival analysis. In particular, we have cited four latest research papers related to our work in Introduction section and cited a latest research paper about survival analysis in Results section. The cited papers in introduction section demonstrate the arising significance of handling multivariate censored time-to-event data while the paper cited in result section indicates the varying result of survival analysis attributed to diverse distributions which our research has handled.

Comments 3. To further elucidate the results and underscore the advantages of CTIVA, it is suggested incorporating more visual demonstrations, particularly when juxtaposing CTIVA with other methods. Graphical representations can offer intuitive insights and make the comparisons more tangible for readers.

Author’s response: We thank the reviewer’s valuable comments to refine the manuscript by incorporating visual demonstration of the proposed CTIVA. We have included the graphical comparison of the raw censored data and data estimated with CTIVA in NCBI dataset with Fig 3 in result section. In the Fig 3, subfigure (a) demonstrates the difference between raw censored data where both event 1 and 2 are censored and data estimated with CTIVA. Additionally, subfigure (b) depicts the ANOVA result of the raw censored data and estimated data with bar graph of average values of interval time. The p-values provided in subfigure (b) are calculated through the ANOVA test.
